# Cocaine

**Published:** 1896-02

**Authors:** J. L. Newborn

**Affiliations:** Memphis, Tenn.


—*Microcosm.*

---

ARTICLE II.

### COCAINE.

BY J. L. NEWBORN, D. D. S., MEMPHIS, TENN.

"Certainly," says an able editorial in the *Cosmos*, "the ghastly record of fatalities consequent upon the use of cocaine preparations is long enough to demand a halt, and that the use of this drug be placed on a clearly defined legal basis."

In two papers published by Dr. Mattison, medical director of the Brooklyn Home for Habitues, on cocain poisoning, covering a period from May 23, 1887, to March 1891, he cites thirteen deaths and nearly two hundred less lethal cases. In another paper, covering a later period, increasing the fatal record, he describes two cases: strong men, stricture; twenty minims of four per cent. solution of cocaine thrown into the urethra—not hypodermically—produced violent convulsions, and in spite of all efforts, death in four minutes. A young woman in the hands of a complexion artist suffered great pain from the application of a strong solution of carbolic acid to the affected part. To relieve this a four per cent. solution of cocaine was freely applied—not hypodermically. In a few minutes she became excited, said she felt strange, walked to the window, and fell dead.

He emphasizes the need of great care in all cases of cocaine anæsthesia by the following pertinent conclusions:

1. Cocaine may be toxic.
2. This effect is not rare.
3. There is a lethal dose of cocaine.
4. This dose is uncertain.
5. Dangerous and deadly results may follow doses usually deemed safe.
6. Toxic effects may be the sequence of doses large or small, in patients young or old, the feeble or the strong.
7. The danger is greatest when given under the skin.
8. Cardiac or renal weakness increases the risk.
9. Purity of drug will not exempt from ill result.
10. Caution is needful under all conditions.
11. Reclu's method, Corning's device, or Esmarch's bandage should be used when injecting.
12. Nitrate of ampl, hypodermic morhpia, hypodermic ether, alcohol, ammonia, and caffein should be at command

History has repeated itself along lethal lines as regards cocaine so often, says the doctor, that it really seems surprising that any one at this day should question its powers for harm. It may not be known to all that cocaine has killed in a smaller dose than morphine, but that is a fact. It may not be known to all that cocaine has killed in a shorter time than morphine, but that too is a fact. "If, then, we have such a narrow margin of safety in the use of cocaine, in accurately known doses, the use of a nostrum containing cocaine, in which the existence of the drug is either denied or is of unknown amount, becomes little short of criminality." In spite of this grewsome record, which is but a fractional per cent. of all its untold, therefore unknown temporary and permanent injurious and often fatal, after effects, yet "fools rush in where angels fear to tread."

I suppose that every State and territory and county and important town from Puget Sound to the Florida Keys, and from Wool-as-took to the Rio Grande, has been canvassed by the nostrum vender, selling to dentists the exclusive rights to use these dangerous cocaine compounds. Our offices are periodically flooded with fancy-colored leaflets offering to give us a half-dozen bottles of their secret "hell fired" stuff if we will send on three dollars for the best hypodermic syringe ever made, and two or three extra needles thrown in. Some of these seductive offers come from men claiming to be dentists For references they often inclose pages of flaming certificates from men with "D.D.S." to their names. Often there are those with an easy conscience who have never learned to say no, but greedy of gain, and eager to double or quadruple their practice, with no proper knowledge of dentistry, ignorant of the most elementary features of the action of cocaine, therefore easy victims of the patent nortrum vender. These do not recognize the danger signals exhibited by distressed nature in the pallid cheek, the clammy brow, the labored breathing, and the wild, anxious stare. With a judicious admixture of "cheek," "gab," red rubber, and a hypodermic syringe loaded with something, they know not what the ignorant public becomes an easy prey, upon whom is entailed, by a wholesale mutilation of the young and old, the loss of useful organs that should have been preserved through life, and a worthless substitute, as a personal souvenir of their stupendous folly.—*Microcosm.*

